# “Mendelian Randomization” Approach in Economic Assessment of Health Conditions

**DOI:** 10.3389/fpubh.2019.00002

**Published:** 2019-02-04

**Authors:** Vipin Gupta, Mohinder P. Sachdeva, Gagandeep Kaur Walia

**Affiliations:** ^1^Department of Anthropology, University of Delhi, New Delhi, India; ^2^Public Health Foundation of India, Gurugram, India

**Keywords:** health economics, Mendelian randomization, genes, epidemiology, public health

## Abstract

The increased prevalence of non-communicable chronic diseases (NCDs) is reflected in the rising economic burden of health conditions. Observational studies conducted in health economics research are detecting associations of NCDs or related risk factors with economic measures like health insurance, economic inequalities, accessibility of jobs, education, annual income, health expenditure, etc. The inferences of such relationships do not prove causation and are limited to associations which are many times influenced by confounding factors and reverse causation. Mendelian randomization (MR) approach is a useful method for exploring causal relations between modifiable risk factors and measures of health economics. The application of MR in economic assessment of health conditions has been started and is producing fruitful results.

## Introduction

The world is facing epidemiological transition that has been attributed the rise of non-communicable chronic diseases (NCDs) and shifted the goal-post from fatal communicable diseases (1). United Nations' Sustainable Development Goals (SDG, 2015) has specified NCDs as one of their important health related targets (Target-3.4) for improving overall wellbeing of human populations (2). NCDs include chronic diseases like cardiovascular disease (CVDs), cancer, diabetes, and chronic respiratory diseases, etc. Global Burden of Disease (GBD) has observed rise in proportion of deaths attributable to NCDs from 57.6% in 1990 to 71.3% in 2015 (3). India spends only 4.5% of the GDP on health (4) and was ranked 143 in SDG index under which the indicator of NCDs (i.e., age-standardized death rate due to cardiovascular disease, cancer, diabetes, and chronic respiratory disease in populations aged 30–70 years, per 100,000 population) was scored low (i.e., 46 less than the median score of 50; scale used: 0 to 100) (2).

Among the South Asian countries, India and Pakistan, had received the bulk of development assistance for health (DAH) which is a financial (or in-kind) resource transferred from development agencies (like UNICEF, WHO, etc.) to low- and middle-income countries (LMICs) primarily for maintaining or improving health (4, 5).

Over 60% of deaths in India are due to NCDs (6) and it was ranked second in the burden of NCDs in 2015 vs. average DAH allocated for 2012–14 (4). NCDs may cost about $4.58 trillion between 2012 and 2030 to India, of which, $2.17 trillion and $1.03 trillion will be due to CVDs and mental health conditions, respectively (7). CVD patients in India spend higher out-of-pocket health expenditure and rely more on borrowing and household asset sales (8). The rising economic costs are due to increased burden of NCDs and their risk factors. For example, alcohol is a known risk factor for several types of cancers and cardiovascular conditions, like hypertension, stroke, etc. (9). Lifestyle based risk factors, like physical inactivity, alcohol abuse, tobacco use, and high body mass index, etc., jointly accounts for 61% of cardiovascular deaths (10).

Health economists are interested in studying the role of socio-economic patterning of health inequalities related to NCDs and their risk factors (11). A study on NCD multi-morbidity and its impact on health care utilization and out-of-pocket expenditure (OOPE) found that medicines constitute the largest proportion of OOPE (12). The scope of research in health economics is gradually widening with the availability of large resources containing variety of data on biological and economic factors. It is allowing the health economists to evaluate variety of economic factors in relation to risk factors of NCDs (13, 14). Epidemiologists in order to balance the distribution of confounders between the analytical sub-groups, usually adjust the statistical models for confounding while evaluating exposure-outcome associations in observational studies. However, it is difficult to authenticate whether statistical adjustment has truly nullified the effect of confounders in reported associations. Testing of such reported associations fail in RCTs. The analytical approach of Mendelian randomization (MR) can be very helpful in preventing failures of RCTs by providing strong causal evidence between exposure and outcomes in comparison to typical observational studies. The objective of this paper is to review the use of Mendelian randomization (MR) approach in studying health economic aspects of NCDs.

## Causality in Epidemiology

The interpretation of association between two factors in an observational study is limited to the plausible relationship and it does not prove causality (15). Epidemiologically, observational studies like cross-sectional population based or hospital based designs are also prone to biases like reverse causation and confounding (16, 17). The science of etiological epidemiology provides triangulation strategies that help in obtaining reliable evidence for translating it into the policy statement. The causal research question can be addressed by triangulation approach which allows the integration of results from different approaches each having unrelated and different sources of potential biases (18). Appropriately designed randomized controlled trials (RCTs), where study participants are randomly assigned to the intervention and control groups, are considered as gold standard in detecting the causal evidence (19). However, the evaluation of long term effects of social behaviors (e.g., health care expenditure, academic performance, supplementary nutrition to the child, alcohol consumption and smoking) on human health may not be feasible to test with RCTs (20). Moreover, high cost of RCTs and feasibility problems due to ethical considerations may pose further challenges in their implementation. Therefore, we need alternative approaches for testing causal relationships in research related to health economics that can overcome the potential biases of observational designs and also provide reliable evidence for causal associations. Mendelian randomization (MR) is one of such approaches whose reliability has been established in epidemiology and is gaining popularity among health economists in testing causal research statements and obtaining consistent evidence in a cost effective manner.

## Mendelian Randomization

MR utilizes genes as an instrument for modifiable exposures in order to test their causal relationship with health outcomes or risk factors (21). The random allocation of groups in MR is based on Mendelian law of independent assortment, i.e., distribution of genetic variants inherited randomly from parental to offspring generation. Thus, the differences between individuals due to genetic variation in MR will not be subject to the confounding or reverse-causation bias which generally distorts the results of observational studies (22).

The random distribution of known and unknown confounders in MR approach is natural in nature which is achieved by genotypes that are known to be associated with specific health condition. Like intention-to-treat in RCTs where study participants are analyzed irrespective of their compliance with the intervention, MR investigates people irrespective of their genotypes that may lead to differences in outcomes (23). Therefore, MR designs are generally considered similar to RCTs. Many times randomization in RCTs is likely to be biased due to the reason of non-compliance. For example, randomization allocates the study participants in RCTs only on the basis of specific treatment chosen. People exercise their choice to reduce high body mass index, through selective lifestyle changes, and that influences their long and short term social outcomes like educational attainment and career achievements. The element of exercising choice while treatment selection by the participants is usually influenced by their socio-economic position, dietary habits, caste and religion, thus, has effects on their level and duration of compliance (24). Unlike observational studies, the genetic variants used in MR are largely unrelated to social and behavioral traits, and avoid systematic biases in randomization process. The strength of MR lies in the use of standard instrumental variable (IV) approach commonly applied in econometrics where exogenous or independent variables are used to detect the effects of endogenous or dependent variables.

**Figure 1:**
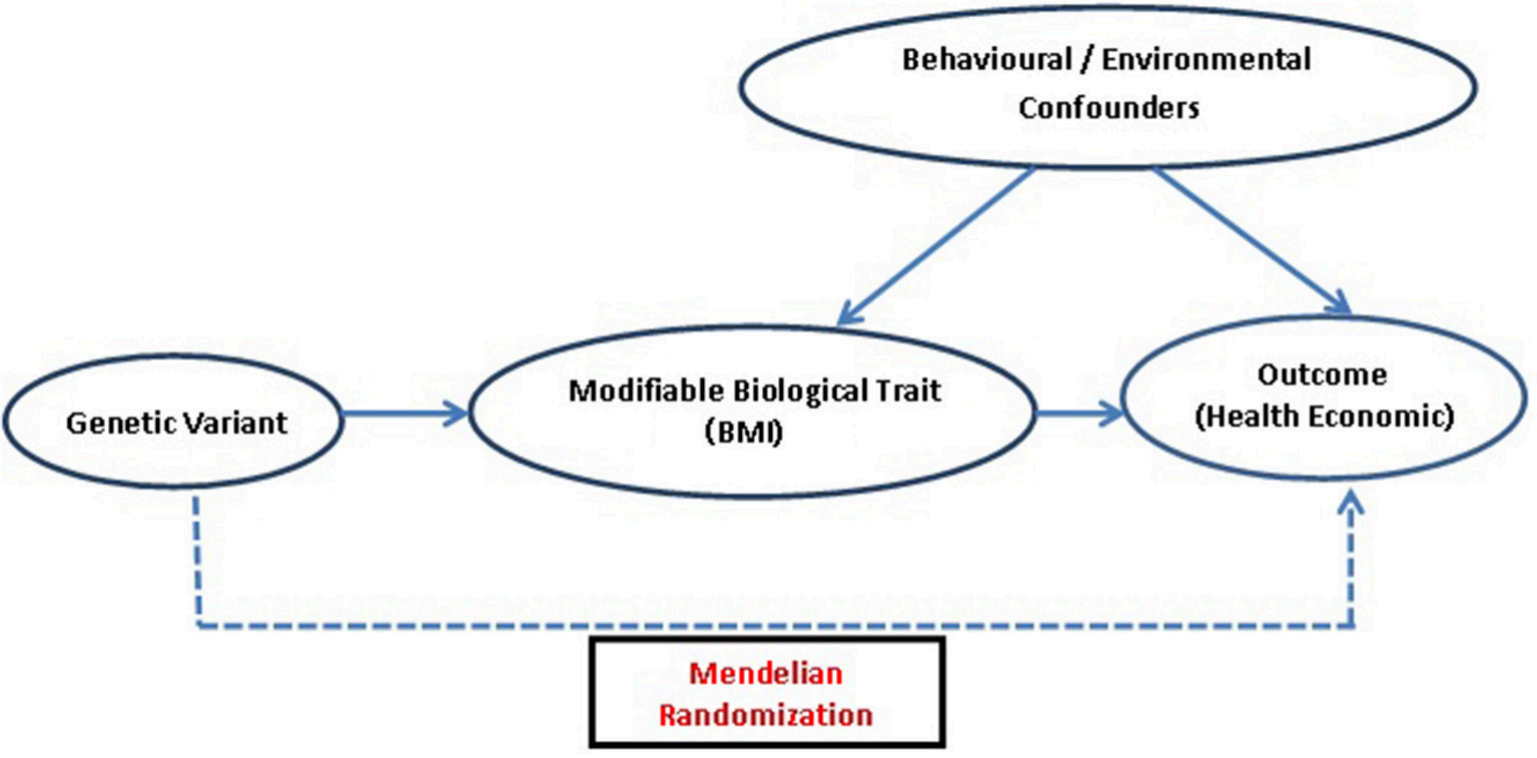
Diagrammatic representation of MR approach: assessing causal association of genetic variant which acts as an IV, i.e., a proxy for modifiable biological traits, with outcomes related to health economics.

The potential genetic variant for IV analysis must comply with three main assumptions of MR (25, 26): (1) the genetic variant must be truly associated with the health condition or its measure, so that it can be used as a proxy for exposure of interest (2) it should not be associated with measured/unmeasured confounders of the exposure-outcome relation, and (3) exclusion restriction criteria, i.e., the instrument/genetic variant should not be directly associated with the outcome and its effect must be mediated only through the exposure, implying that no alternative causal pathway exists between instrument and outcome except via exposure (27). These assumptions are highly credible when the effects of an exposure are directly due to the genetic variant (Figure 1).

MR assumptions lose credibility to some extent when exposures are polygenic traits i.e., multiple genetic variants may be responsible for the exposure. The multiple genetic variants used for polygenetic traits may lead to pleiotropy i.e., a single gene having multiple expressions, by being involved in multiple pathways (27, 28). This limitation of MR can be statistically resolved using MR-Egger method (27). MR approach has been widely utilized in the last decade and various methods have been proposed to improve the inferences given the potential limitations (29).

One of the important step of the MR is to rely on a single genetic instrument which is used as a proxy for the modifiable exposure. This can be partially addressed by using multiple variants and using weighted allelic score as an instrument (25). It increases the proportion of variance explained by the instrument, thus increasing power of the study (30), apart from addressing the other issue like pleiotropy or being in linkage disequilibrium (LD) or in close proximity with a variant that affects the outcome (23). If a genetic variant has a pleiotropic in nature (i.e., multiple effects) or is in LD with another gene, it cannot represent a valid instrument for the exposure of interest, as it may produce biased findings. Over the years, MR has methodologically moved forward from the use of simple IVs based on single variant to the composite IVs based on multiple genetic variants without the knowledge of their functions (26). Recently, method for conducting pleiotropy robust MR has been proposed which first estimates the amount of pleiotropy in the model and then corrects it (31). Bi-directional MR approach has also been suggested to be useful in understanding causal pathways where genetic instruments for both exposure and outcome are applied in both directions in MR analysis to establish the direction of causal pathway (23).

## Guide for MR

The identification of genetic instrument variable (IV) to be used as proxies for exposures in MR analyses should satisfy the underlying assumptions of an IV method.

### IV Is Associated With the Exposure (*Relevance Assumption)*

Relevance means the genetic variant(s) that can be used as an instrument that robustly predicts the exposure. In order to identify genetic variants associated with the exposure, we need to conduct a comprehensive literature search (using PubMeD and MEDLINE) for articles based on genome-wide association studies (GWASs) directly related to the exposure. Nowadays, there is an online GWAS catalog of published literature available (https://www.ebi.ac.uk/gwas/) for identifying genetic variants associated with exposure of interest. “PhenoScanner” is another online resource developed by University of Cambridge, which can be used for genotypic-phenotypic associations (32). These resources are helpful in developing a list of potential genetic variants associated with the exposure (for using them as instruments). One can extract the gene names, single nucleotide polymorphisms (SNPs), their effect sizes (along with their direction and standard errors), biological pathways and significance level (*p*-values < 10^−8^) from the original GWAS articles. This list further guides the second round of literature search for identifying the primarily identified variants that have been replicated and validated in other populations to prepare a list of well-established genetic variants. Researcher should give more weightage to those genetic variants which are well replicated and validated in the target population of interest, having strong association with the exposure, and have relatively large effect sizes (explaining the maximum variation in the exposure). In case of unavailability of reliable instruments, one can also use multiple SNPs to generate a “risk score” (a polygenic score) which can be used as an instrument in MR studies instead of using one single SNP.

However, it is possible that one may not be able to find a GWAS nor a validation study related to the exposure in their target population during literature search. Therefore, the last step would be to genotype the potential genetic variants in a statistically powered sub-sample to ensure the genetic association with the exposure in the desired population. It should be noted that the instruments should not be internally derived using single sample to avoid bias (i.e., Winner's curse: the overestimation of association between SNP and exposure) (33). Therefore, it is advised that the identification of an instrument and MR analyses should be performed in different set of samples from the same population.

For MR analyses one can use *R package* (i.e., TwoSampleMR) available at “MR-Base” website which is also a database of genotypic-phenotypic associations based on GWAS studies (34). *MRrobust* is a STATA package for MR analyses (35).

### IV Is Not Associated With Any Confounder of the Exposure–Outcome Association (*Independence or Exchangeability Assumption)*

The MR designs are considered similar to RCTs and the key property of RCTs is that the two randomized subgroups are exchangeable in nature which means the expected distribution of confounders is balanced for both the subgroups. Similarly, in MR the division of population into genetic subgroups based on IV (i.e., genetic variant) leads to exchangeable subgroups, independent of all observed and unobserved confounders, having similar distribution of confounders like RCTs. However, this assumption cannot be tested empirically. Nevertheless, the genetic variant to be used as instrument should not be directly associated with any of the possible confounders affecting the relationship between exposure and outcome of interest. Moreover, one can assess the biological mechanisms (given in Table 1) that can make a genetic variant unsuitable for IV.

**Table 1:** Types of biological mechanisms that can violate IV assumptions and their possible solutions.

**Biological mechanisms**	**Definition**	**Mechanism of IV assumption violation and their solutions**
Pleiotropy	It refers to the phenomenon of association of one genetic variant with multiple traits or outcomes or risk factors (36). In biological pleiotropy an instrument will affect both exposure and outcome through different pathways. For example, independent GWASs have found several cross phenotype effects, especially for autoimmune diseases and psychiatric trait (37)	IV assumption is violated if a genetic variant is associated with another risk factor of the outcome; or directly with the outcome itselfOne can use multiple genetic variants (polygenic score) or can use *cis* variant which may not have pleiotropic effects. The use of polygenic score may also have some limitations due to their potential correlations with confounders therefore may violate MR assumptions. This problem can be avoided by integrating the polygenic scores within family based design (38). The relevance of no pleiotropy assumption in MR decreases when the shared genetic etiology due to genetic correlations is present between two phenotypes suggesting common etiology. Such regions scan be identified using colocalization methods, i.e., same locus is independently associated with two phenotypes (39), will be useful for gathering evidence either in favor of pleiotropy or causality between phenotypes with directions of associations (40). Phenome-wide associations, where genetic variants are assessed to find their associations with multiple phenotypes, can be used to detect the probable regions of genetic correlations (41).*MR-PRESSO* is a R package used for assessing pleiotropy in multiple SNPs MR (42).
Canalization	An adaptive compensatory phenomenon during development that may modify a phenotypic response to genetic change to the extent of its reduction or complete absence. It leads to the expression of a gene function through different biological pathway. It may lead to the accumulation of cryptic genetic variations which generate heritable genetic variations in a population (43).	It may have downstream effects on other variables which may lead to differences between groups with respect to exposures as well as outcomes, thus violates IV assumption.No straight solution is available. One can observe during the life course of an individual, as per the timing of expression, to find whether canalization is a problem or not.
Linkage disequilibrium (LD)	It is the statistical associations between alleles at different loci (44) i.e., when two genetic variants on the same chromosome (genomic regions) are physically close to each other and inherited together.	IV assumption is violated if the considered genetic variants is in close proximity (i.e., in LD which leads to the correlation in the distribution of two genetic variants) to another un-genotyped variants associated with other risk factor or outcome.Populations with different LD structure should be used for MR
Effect modification	A term used when the effect size of an exposure or treatment on an outcome differs among the groups of patients/participants with different characteristic may be due to statistical interaction between the exposure and a covariate (45).	IV assumption is violated because the causal effect of the exposure will change across the levels of covariate
Population stratification	When multiple distinct ethnic groups having different ancestry are there in the study sample that can lead to spurious association due to varying frequency of genetic variants and distribution of exposure in subpopulations. Such associations are just due to different subpopulations or systematic ancestry differences and not due to genetic variant (46).	Instrument selected for MR based on spurious genetic associations will violate IV assumption.Restrict the MR analyses to homogenous populations having common ethnic background. Or assess the population stratification using statistical methods, like, Principal Component analysis.

### IV Does Not Affect the Outcome, Except Possibly via Its Association With the Exposure (*Exclusion Restriction Assumption*)

The genetic instrument should be associated with the outcome only through the route of exposure, and not directly. The conditional assumption of exclusion restriction can be evaluated through the knowledge of biological pathway of the genetic instrument used (47). Since the specific functions of the genetic variants and their exact biological mechanisms are generally unknown, the direct assessment of exclusion restriction assumption is not possible, thus, validity of this “exclusion restriction” assumption cannot be measured. The biological mechanisms like canalization and pleiotropy can also violate exclusion restriction (48). One can conduct gene by environment interaction analyses that could provide evidence for canalization (48). A pleiotropy robust Mendelian randomization (PRMR) method has been suggested which corrects for pleiotropy after estimating the magnitude of pleiotropy (31). Moreover, if it is difficult to estimate the degree of pleiotropy even then PRMR can be applied for the sensitivity analyses. Recently, researchers have suggested an alternative approach, i.e., genetic instrumental variable regression, which controls for pleiotropic effects using polygenic scores (49). Furthermore, a new way to estimate IV (i.e., MR G-Estimation under No Interaction with Unmeasured Selection) which is robust to additive unmeasured confounding and exclusion restriction violations has also been suggested (50).

## MR in Health Economics and Insurance

The estimation of healthcare cost expenditures of medical conditions are important for planning public health programmes. Many times confounding factors, like socio-economic status, natural histories of diseases, ascertainment bias and measurement error make the economic assessment of health outcomes difficult in observational studies (48). Moreover, exposing patients to interventions just for collecting information on cost consequences of health in RCTs is also not practical and ethical (51). Since genetic variants associated with health conditions are generally unrelated to known and unknown confounders (i.e., environmental and behavioral factors), therefore, MR designs offer the reliable detection of causal effects of health conditions and thereby the cost of healthcare.

**Case Study**

Scholder et al. (57) examined causal association between child's/adolescent's height and cognitive, mental health and behavioral outcomes like academic performance, IQ, self-esteem, depression symptoms and behavioral problems, using genetic determinants of height utilizing Instrument variable analyses using available data from Avon Longitudinal Study of Parents and Children (ALSPAC).

Authors used a set of nine genetic variants associated with height among Europeans: *HMGA2* (rs1042725), *ZBTB38* (rs6440003), *GDF5* (rs6060373), *LOC387103* (rs4549631), *EFEMP1* (rs3791675), *SCMH1* (rs6686842), *ADAMTSL3* (rs10906982), *DYM* (rs8099594), and *C6orf106* (rs2814993). Firstly, authors tested the validity of genetic variants to be used as instruments for height by testing all the MR assumptions. The genetic instruments were found to be uncorrelated with a large set of family background variables which my confound the relationship between height and outcomes of interest. Authors also ran two falsification checks. They first examined the effect of height on maternal education for which they did not assumed any effect. Thereafter, the effect of height on body weight was examined for which they assumed a strong effect. After confirming the validity of instruments they examined the causal relationship between height and set of cognitive, behavioral and mental health outcomes.

The IV results showed that there is causal relationship between taller height and cognitive function among girls with respect to national exam taken at age of 14 years (β = 0.54, SE = 0.24) and IQ test at the age of 8 years (β = 0.67, SE = 0.28). They did not find any evidence of height effecting scholastic self-esteem, self worthself-worth, or depression. Authors suggested that height presents disadvantage rather than advantage as it was fund to increase hyperactive behaviour among girls and emotional and peer problems among boys.

**Advantages of MR**

- Highly useful in strengthening the evidence from purely observational to causality which can effectively paves a path for RCTs.
- MR also useful in detecting the direction of causality of association between the exposure and outcome.
- Use of genetic variants as proxy for exposure helps in the random allocation of confounders between exposed and unexposed that also eliminates selection bias. Moreover, the genetic data can be robustly generated without errors at affordable cost that tend to decline more in future.
- Methodology for conducting MR analysis is well established due to the continuous efforts made by the researchers, and it has the potential to grow further in future.
- Identification of genetic instruments from available databases is useful and manageable even for researchers, having no background in genetics, with little training.

**Limitations of MR**

- The inferences based on modeling driven MR approach are heavily dependent upon the credibility of the assumptions underlying MR.
- The execution of MR is contingent upon the identification of genetic proxy for the exposure of our interest.
- Large sample sizes are required for MR analysis, due to small effect sizes of genetic variants to meet the optimum statistical power, which is difficult to obtain in low and middle income countries.
- Genetic instruments of MR are selected from hypothesis free genome-wide association studies which lacks in-depth understanding of the mechanisms of associations between genetic variants and traits/diseases.

The health economists are increasingly interested in studying the causal effects of modifiable non-genetic factors on the outcomes of their interest (Table 2). For example, a study on the effect of prenatal alcohol exposure on child's educational outcome in terms of academic achievements has been reported (52). Since the feasibility of studying fetal blood alcohol levels is very low and may pose some ethical challenges, they examined the data on maternal alcohol exposure during pregnancy as a proxy for alcohol exposure *in utero*; the results were ambiguous due to confounding of socio-economic position. Therefore, MR approach was applied by using *ADH1B* gene as an instrument variable for maternal alcohol exposure and found long term negative effects on child's educational achievements (52). Another study on longevity and frailty (i.e., a state of high vulnerability to trivial stressors) did not find any causal association of triglyceride levels with these two complimentary aspects of aging. This study also rejected the previous observational associations of triglyceride levels with longevity and frailty (53). Such studies can be attempted again using MR approach with the help of established genetic markers of triglyceride levels.

**Table 2:** Summary of MR studies related to health economics and their findings.

**Populations**	exposures gene [SNP^*^]	**Outcomes**	**Results**	**References**
British	Maternal alcohol intake (i.e., *in utero* alcohol exposure) *ADH1B* [rs1229984]	Child's Academic Achievement	Negative effects of parental alcohol exposure on child's academic achievements	(52)
Chinese	Triglyceride levels *APOA5* [rs662799]	Longevity and Frailty	Triglyceride levels are not causally association with longevity and frailty	(53)
British	Child's Body mass index *FTO* [rs9939609] and *MC4R* [rs17782313]	Child's Academic achievement	Higher adiposity is causally associated with poor academic performance	(54)
British	Height and Body mass index *Genetic risk score* was estimated for height and BMI [based on 396 SNPs related to height and 69 SNPs related to BMI]	Socio-economic status	Higher height and body mass index are causally associated with lower annual household income of both men and women.	(55)
Finnish	Stature Genetic risk score was estimated (based on 180 SNPs related to stature)	Labor market outcomes: Earnings and labor market attachment from 2001 to 2012 (Earning-average salary Attachment-average employment)	No causal association was observed between height and labor market outcomes (may be due to less sample size)	(56)

* *SNP, single nucleotide polymorphism (a type of genetic variant)*.

Observational studies have shown that taller stature and low body mass index (BMI) are associated with high SES in developed countries (58, 59). The relationship between child's height and economic outcomes is confounded by a variety of detrimental environmental experiences during childhood, like, unobserved family income and variation in their nutrition. With the help of MR approach a study assessed the causal effects of height on human capital accumulation (in terms of academic performance, IQ, self-esteem, depression symptoms and behavioral problems) (54). They used genetic variants as proxy of height un-confounded by above environmental experiences and found an important role of height in child's cognitive performance, handling emotional and peer pressures. Recently, a large MR study (119,000 participants of UK Biobank) was conducted to examine the causal relationship between stature, BMI and SES using genetic variants as unconfounded proxies for height and BMI (55). It was reported that taller stature (of 6.3 cm) leads to higher educational attainment that further leads to odds of 1.12 for higher status of jobs and higher annual household income (of £1130). Their analyses also showed a causal association of higher BMI (of 4.6 kg/m^2^) with lower annual household income (~ £2940) (55).

Physical traits and economic success are inextricably entwined through body size, beauty and height (59–61). A long term empirically tested belief suggests that taller people have better cognitive and non-cognitive skills, thus, possess more positive qualities which helps them to earn more than relatively shorter people (59, 61). The causal role of height on earnings was evaluated in a study using twins to attenuate the influence of genetic factors and family background (62). They reported no association may be due to the inability of twin design to eliminate the confounding from early life conditions and differential parental investments (62). A recent study applied MR using gene score of previously associated genetic variants of height as a proxy or IV to examine its relationship with administrative information related to long term labor market outcomes i.e., earnings and labor market attachment (56). Their MR analysis did not find any association of gene score with the earnings, may be due to low power to conduct MR with sample size of 2000 individuals, and confirmed the findings of the above mentioned twin study (which are generally difficult to conduct). In their opinion, the previously reported associations may not be causal as they have observed (56) but it is difficult to conclude unless proved with the help large sample size to get more precise estimates.

Further, education is one of the three components of a regular instrument for assessing socio-economic position. In the above study, protective causal effects of education on obesity in Finnish population using MR design was reported (56). It was suggested that low birth weight is associated with lower educational attainment and earnings (63). MR based economic evaluations in future can assess the short and long term causal relationships between birth weight and educational attainment and earnings. The medical reimbursement is a central aspect of insurance for the health insurance companies especially for medical technologies which rely heavily upon their clinical efficacy (64). MR can be used as a cost and time effective strategy for testing the clinical efficacy of medical technologies instead of costly RCTs (65).

## Conclusion

MR has high potential in research related to health economics and health insurance consequences, and will be very useful in making informed decisions with respect to policy coverage on latest medical technologies. The utility of MR is even higher in countries with limited resources for health care financing and moving toward universal health coverage as an important public health policy.

## Author Contributions

GW, VG, and MS conceptualized the idea of the manuscript. VG and GW completed the literature search. GW and VG wrote the manuscript. MS critically reviewed and improved the manuscript.

### Conflict of Interest Statement

The authors declare that the research was conducted in the absence of any commercial or financial relationships that could be construed as a potential conflict of interest.
